# Normative Data and Reliability of the Moving Shapes Paradigm

**Published:** 2020-04

**Authors:** Zahra Shahrivar, Mehdi Tehrani-Doost, Anahita Khorrami Banaraki, Azar Mohammadzadeh

**Affiliations:** 1 Department of Psychiatry, Roozbeh Hospital, Tehran University of Medical Sciences, Tehran, Iran.; 2 Brain and Cognition Clinic, Institute for Cognitive Science Studies, Tehran, Iran.; 3 Research Center for Cognitive and Behavioral Sciences, Roozbeh Hospital, Tehran University of Medical Sciences, Tehran, Iran.

**Keywords:** *Children*, *Moving Shapes Paradigm*, *Norm*, *Reliability*

## Abstract

**Objective:** Moving Shapes paradigm is a test that evaluates intentionality as a theory of mind (ToM) component. This study aimed to assess the normative data and reliability of this test in a community sample of 9-11-year-old children.

**Method** **:** A total of 398 children aged between 9 and 11 years were recruited from mainstream elementary schools through a random cluster sampling. All participants were evaluated using the Moving Shapes paradigm. To evaluate test-retest reliability, the test was administered again after 2-4 weeks.

**Results: **The intentionality mean score was 29.70 (+5.88) out of 60. There was no significant difference between girls and boys in test scores. Age was not significantly related to the paradigm variables scores. Ten percent of the participants achieved the scores below 22, and 10% above 37. Cronbach’s Alfa was 0.40 for the intentionality score. The test-retest reliability was fair to good (0.43 - 0.79) for different groups of animations. The inter-rater agreement was 80%.

**Conclusion: **The study found that the Moving shapes paradigm is a reliable instrument to evaluate intentionality in normal school-aged children**.**

Generally, humans are able to relate to people through the development of metacognitive processes, including mentalizing and theory of mind (ToM) abilities, which are the basic skills to regulate communication and interaction. ToM is a term, which has been introduced first by Premack and Woodruff (1978), describing chimpanzees’ ability to understand others’ minds. ToM refers to realizing individuals’ thoughts, beliefs, and intentions, enabling us to infer others’ behavior to their internal mental states (2). Mentalizing focuses on reflection of affective mental states. It leads to understanding others’ feelings, desires, and intuitions (3). 

Many measures have been developed to assess different aspects of ToM in children and adults. The primary tests (4) were stories focused on first- and second-order false belief tasks. These tests assess an individual’s ability to realize that other people may have different beliefs and opinions (first order: what someone thinks). 

Moreover, these tests evaluate whether the examinee realizes the sequential process of people’s mind-reading (second order: what someone thinks about another person’s content of thought). Individuals were presented with some questions about the story dolls and props to show if they understand the mental states of those characters. To reduce the difficulty of the children’s understanding of beliefs, Zaitchik’s developed the false photograph test (1990). Tier 1 and Tier 2 terms are common in theory tests, and children of different ages provide different answers to them (2). 

Next generation of ToM tests, including nonverbal instruments, assess higher levels of mind-reading ability; for example, realizing intentionality, sarcasm, lies, bluffs, and emotions. Some samples of these tools are Gallagher’s cartoon task test (2000), Baron Cohen reading in the eyes test (2001), and Egeth and Kurzban’s meta photograph test (2009). 

In addition, more dynamic tests (9) have been produced such as movie clips showing natural or acting behavior in a social situation to take account of the mental states of the characters. These visual tests also need some verbal skills.

One of the main aspects of ToM is intentionality, which refers to the attribution of goals, beliefs, and desires as the mental causes of behavior (10). Human beings understand intentionality from different stimuli such as facial expression, prosody of speech, body movements, and content of communication. It has been found that properties of the motion, instead of the characters’ appearance, are more influential in perceiving intentionality (11 & 12). Three-month infants discriminate animated motions from mechanical ones (13). By the end of the first year of life, infants can understand that intentional states underlie the movements and expressions of others, meaning that infants appreciate agents as intentional actors (E.g. a mother gives a doll to her baby.) and as intentional experiencers (E.g. a mother experiences the state of her love (desires) and joy (emotion) to play with her baby.) (14). 

Heider and Simmel (1994) were the first to show this concept in their famous study. They used a film showing a rectangle containing 2 triangles and a circle moving around. They found that the examinees inferred intentions to the moving shapes and realized that animated motions can be perceived as intentional actions. Other versions of animations and films had been used by other authors (16, 17 & 18). Castelli et al (2000) developed 3 different types of animations using 2 triangles without any vocal or facial expression cues. They used 3 conditions: random movement, goal-directed interactions, and ToM interactions. The moving shapes paradigm uses geometric patterns to examine the inference of mentalizing and is based on motion detection. It does not need emotion recognition ability. Since intentional understanding develops before the age of 1, it seems that this paradigm is less dependent on the development of executive functioning. Compared to the animations, the false belief tasks are dependent on executing function ability, including inhibitory control. It seems that under 3-year-old healthy children cannot pass false belief tasks due to their inability to inhibit responses.

Given the importance of intentionality in the assessment of the ToM development, the use of the moving shapes paradigm as a test not dependent on literacy can be a good tool in this field. There is a need to use a valid and reliable test to evaluate intentionality in the population of Iranian children. Some parts of the moving shapes paradigm developed by Castelli et al (2000) have been used in preliminary research (20) on a small sample of healthy developing school-aged children in Iran. The authors reported similar results to the findings of other studies in terms of children’s understanding of people’s intentionality, use of emotional words, and accuracy, and length of phrases. This study aimed to assess the psychometric properties and normative data of all parts (total number of 12 animations from the same paradigm) of the moving shapes paradigm as a major task of evaluating intentionality in a large sample of healthy children. This study was conducted as a part of a larger research on evaluating ToM abilities in a community sample of school-aged children in Iran (21). We reported different validity types of the paradigm in another paper (22). 

## Materials and Methods

***Participants***

The statistical population included all students studying at grades 3-5 mainstream schools of central parts of Tehran. The research sample was the children who agreed to participate in the study and their parents. All students had normal intellectual abilities based on their academic and parents’ reports. Hence, the inclusion criteria were normal IQ and being at grades 3-5. Since the main study was performed using the strange stories test, which is dependent on children’s level of literacy and text comprehension, the participants were selected from third to fifth grades. Among the total number of 481 students approached, 83% participated in the research (girls = 50.8%). All participants completed the study without any attrition. The mean (standard deviation) of their age was 9.96 (0.916) years. 

***Moving Shapes Paradigm***

The paradigm used in this study was reproduced using the Macromedia Flash version 9 based on the animations developed by Castelli et al (2000). After receiving permission from the main developers, the scoring method provided by Uta Frith (personal communication) was translated into Farsi. Each animation consists of 2 characters represented by 2 colored triangles moving around a rectangle. The animations were grouped as random, goal-directed, and mentalizing; each group consisted of 4 animations that were displayed counterbalanced. In the random group of animations, the triangles move randomly without presenting any goals. While in the goal-directed scripts, movements of the triangles represent a goal that can be understood from the sequence of the movements, including fighting, dancing, chasing, and leading. In the mentalizing group, a mental state is depicted from the moves of triangles. The targets of the mentalizing videos were coaxing, surprising, mocking, and seducing.

Children were asked to sit on a chair in a 70 cm distance of a laptop monitor and informed that the room light would decrease so that they could see more clearly when watching the animations. After ensuring that the child was sitting in a relaxed position and having an appropriate vision, they were asked to watch the cartoons and answer the related questions asked by the trained psychologist. Regarding the random animations, the questions were about what the children were watching and what the characters were doing. With regards to the goal-directed animations, the participants were instructed to assume that the triangles were animals or humans. Then, the psychologists asked 2 questions: (a) who are they, and (b) what are they doing? The items were scored from 0 to 2. For each mentalizing animation, some questions consisted of the event, and the character’s actions, their feelings, the reasons for their actions, and the consequences of their interactions. Based on the answers to the 3 main questions, capturing the understanding of intentionality, the scores were calculated. Regarding the degree of appreciation of mental states (19), each item was scored between 1 and 5. Therefore, the maximum total score was 15 for each video and 60 for the 4 mentalizing animations. The scales derived from summing scores were as follow: 1-general rule (GR) = correct description of story sequencing, 2- intentionality score (IN) = degree of appreciation of mental states, 3-appropriateness score (AP) = correct using words and sentences to describe intentionality, 4- length of phrase score (LPh) = length of phrases to describe an animation, 5- number of length score(NL) = number of phrases to explain an animation, 6-emotional terms (ET) = number of emotional terms used to describe the animations, and 7-mental states terms (MT) = number of mentalizing phrases used to explain the animation.

To reduce the influence of confounding factors, including time and place of the assessment, the evaluations were done in a private and silent room at each school from 9-12 o’clock with the same distance from the monitor. The children were not cued or introduced any mental or emotional terms by the examiner to recognize the targets of the conditions. To prevent possible distraction, the answers were recorded. Moreover, the examiners’ related confounding effects were controlled through performing the test by the trained examiners who had high inter-reliability rate in conducting the paradigm.

***Ishihara Test***

Since the moving shapes were in 2 colors of red and blue, and color-blindness would disturb recognizing the movements of colored shapes, the Ishihara test (22) was used to check color detection ability in the participants. This is an efficient test that screens for the red-green color deficiency (23). 

***Procedure***

This study was a psychometric research on a population of normal children. The sample size was calculated based on the formula used for evaluating the mean population. In this formula, the standard deviation is considered as 0.5, with the confidence interval of 95%, and the margin of error of 0.05. As a result, the estimated sample size was 384. To reach this number, 481 students were approached, of whom 399 participated in the study. 

After acquiring permission from the Ministry of Education, the schools in the central parts of the city were selected based on a random clustering sampling. The aims and stages of the study were explained to the schools’ principals. In each school, 2 students at each grade were selected randomly using the alphabetical checklist of the students’ family names. Invitation letters were sent to the students’ home asking the parents to come to the school. Then, the parents were informed about the study. After obtaining consent, they were asked to complete the questionnaires, including demographic forms. The children were invited to do the moving shapes paradigm test in a silent room in the morning. The task was being administered by the psychologists who had been trained in a 2-day workshop to become familiar with the measures performance and scoring method. Interrater reliability was assessed during the training. When the agreement among the raters reached to 80%, they were considered eligible for performing the task. To monitor the raters’ performance, they were supervised by the main investigators. To check test-retest reliability, the test was performed again for one-fifth of the students after a 14-28day interval.

***Analysis***

Using the SPSS software 18, means and standard deviations were calculated based on descriptive analysis. Reliabilities were assessed using the Cronbach's Alpha coefficient and the split-half method. Test-retest reliability was calculated using 2 methods: Pearson correlation and ICC (intra class correlation). A multiway ANOVA was used to find the differences among the academic grades in terms of the paradigm mean differences. A linear regression analysis was performed to assess the association of the ToM results with age. After checking the assumptions, a repeated measure analysis of variance was used to assess the differences between the 4 types of mentalizing animations. Then, the Bonferroni pairwise comparison was done to compare the 2 types of animations with each other. 

## Results

Means and SDs of the moving shapes paradigm scores are shown in Table 1. In addition, a comparison was done between the scores of girls and boys.

As the table shows, there were no significant differences in terms of gender, except for the appropriateness of sentences (AP) (t = -2.639, p = 0.009). With regards to academic grade, the results of multiways ANOVA did not show any significant differences in paradigm mean differences among children in grades 3 (n =136; 34.2%), 4 (n = 129; 32.4%), and 5 (n = 133; 33.4%). Moreover, a regression analysis was done to find if age could predict the ability of understanding intentions depicted in the shapes presented. The results showed that age did not predict intentionality scores (IN) in the participants.

Table 2 compares the major variables scores of the mentalizing group of animations. The results of a repeated measure analysis of variance showed that all differences among the 4 types of mentalizing animations were significant. Based on the Bonferroni pair wised comparison, the participants’ IN scores in “seducing” were significantly higher than the other animations (p < 0.05). IN scores in each of “mocking” and “surprising” were significantly more than “coaxing” (p = 0.000). The ET scores of the “mocking” and “surprising” were higher compared to the “coaxing” (p = 0.003). Similar results were found regarding the MT (p = 0.000). 

To evaluate the normative data, the percentile frequency of the moving shapes paradigm scores were conducted (Table 3). The analysis was performed to find a cut point for the most important variables, including intentionality, mental state terms, and emotional terms. The results showed that 90% of the participants achieved IN scores higher than 22. Furthermore, 90% of the participants used at least one term to describe emotional or mentalizing targets. 

To test the internal consistency for the moving shapes paradigm, Cronbach’s Alfa was calculated across the 4 mentalizing animations groups and the coefficient was found to be 0.35. To find whether the 4 ToM videos could lead to similar scores across the 7 scales, the Alfa coefficients for the test variables were found as follow: NL (0.80), LPh (0.76), ET (0.58), AS (0.58), MT (0.43), GR (0.42), and IN (0.40). Table 4 shows that test-retest reliability coefficients were significant for all variables. The lowest correlation coefficients belonged to IN and MT, while the ET and the NL had the highest correlations. Moreover, ICC coefficients were consistent with these findings. Also, the frequency percentage of the lowest and highest scores of the responders was calculated and no floor or ceiling effect for the test variables was found.

**Table 1:** Means and SDs of Mentalizing Animations Scores in the Total Population, Comparing Boys and Girls

**Moving Shapes ** **Paradigm**	**Means** **and SDs**	**Min-Max**	**Boys** **(N =196)**		**Girls** **(N = 202)**		**T**	**p**
			**M**	**SD**	**M**	**SD**		
GR	7.41+4.113	0-18	7.03	4.388	7.79	3.801	-1.848	0.065
IN	29.70+5.879	12-44	29.21	6.009	30.18	5.724	-1.648	0.100
AP	17.33+5.225	1-34	16.63	5.270	18.00	5.104	-2.639	0.009
LPh	29.11+6.299	13-48	28.94	6.172	29.28	6.432	-0.527	0.598
NL	116.32+55.399	23-498	118.21	58.174	114.49	52.648	0.669	0.504
ET	5.03+3.356	0-24	4.78	3.288	5.28	3.411	-1.508	0.132
MT	4.47+2.951	0-16	4.31	2.930	4.63	2.970	-1.107	0.269

General Rule (GR), Intentionality Score (IN), Appropriate Score (AP), Length of Phrase Score (LPh), Number of Length Score (NL), Emotional Terms (ET), Mental States Terms (MT)

**Table 2:** Comparison of the Participants’ Scores in Each Mentalizing Animation

**Mentalizing Animation**	**IN** **Mean and SD**	**ET** **Mean and SD**	**MT** **Mean and SD**
Coaxing	6.65+2.319	0.94+0.92	0.7+1.00
Surprising	7.52+2.466	1.28+1.31	1.01+1.20
Mocking	7.53+2.590	1.25+1.15	1.18+1.23
Seducing	8.00+2.453	1.56+1.57	1.59+1.38
	p = 0.000 F (3, 1191) = 24.338	p = 0.000 F (3, 1188) = 21.781	p = 0.000 F (3, 1188) = 43.874

IN: Intentionality Score; ET: Emotional Terms; MT: Mental States Terms

**Table 3:** Percentile Frequencies of the Moving Shapes Paradigm Scores (N = 398)

**Moving Shapes Paradigm Scores**			**Percentage**
**IN**	**MT**	**ET**	
22.00	1.00	1.00	10
24.80	2.00	2.00	20
27.00	3.00	3.00	30
28.00	3.00	4.00	40
30.00	4.00	5.00	50
31.00	5.00	6.00	60
33.00	5.00	6.00	70
35.00	7.00	7.00	80
37.00	8.10	9.00	90

IN: Intentionality Score; ET: Emotional Terms; MT: Mental States Terms

**Table 4:** Test-Retest Reliability Coefficients for the Moving Shapes Paradigm (N = 66)

Variable	R	P	ICC coefficient	P
GR	0.651	0.001	0.647	0.000
IN	0.430	0.001	0.427	0.000
AP	0. 658	0.001	0.658	0.000
LPh	0.592	0.001	0.589	0.000
NL	0.715	0.001	0.715	0.000
ET	0.791	0.001	0.791	0.000
MT	0.494	0.001	0.486	0.000

General Rule (GR), Intentionality Score (IN), Appropriate Score (AP), Length of Phrase Score (LPh), Number of Length Score (NL), Emotional Terms (ET), Mental States Terms (MT), Confidence interval:95%

## Discussion

This study was conducted on a community sample of Iranian 9-11-year-old children to evaluate the reliability and normative data of the moving shapes paradigm. As a ToM test, this measure addresses the understanding intentionality of others’ behaviors. The findings of this study showed poor to good reliability of the test.

The majority of ToM tests, including the moving shapes paradigm, have been used in clinical populations with developmental disorders, compared to normal developing individuals (Abell F., Happe F., & Frith U. (2000) & Castelli F., Happe F., Frith U., & Frith C. (2000) Castelli F., Frith C., Happe F., & Frith U. (2002). To our knowledge, this was the first community study that assessed reliability, means, and distribution of the test scores in children. 

In this study, the internal consistency based on the Cronbach’s Alpha was good for the number and length of sentence scores. However, these results were acceptable for emotional terms score and poor for the variables of intentionality, mental state terms, and general rule. To interpret these results, it should be considered that the respondents were free to use different sentences to answer the questions. The deviation of the answers was large, which could reduce the internal consistency of the paradigm. Test-retest reliability based on ICC coefficients were acceptable to good for all variables except for intentionality and mental states terms. The only findings on the reliability of this test were reported by Castelli et al, in which interrater agreement was 65% (19) and more than 90% (24). This coefficient was 80% in the present study, and thus this measure can be used in different time stages.

The mental group of animations consisted of 4 targets, including coaxing, surprising, mocking, and seducing. There was a significant difference among the intentionality (IN) scores of these animations and the mental state terms and emotional terms. Children achieved the highest scores in these variables in seducing animation. It seems that children’s abilities to comprehend animacy are different based on the targets of the videos in the paradigm. This can be interpreted by 2 explanations. First, children achieve the ability to understand seducing better than the other mental state targets. Second, the difference may be related to the clarification and elaboration of the animations which can affect the comprehension of the concept behind the videos. 

The major variables, including IN, ET, and MT were not significantly different between boys and girls in this study. Inconsistent with our findings, Knickmayer et al showed (2006) that normal developing girls used more emotional state terms and achieved higher intentionality scores compared to boys. In several studies, other components of ToM have also been found to be better in girls compared to boys (26-29). Interestingly, in this study, in the same sample who performed the strange stories test (21), the results were higher for girls compared to boys, showing better scores in understanding mental states from the text. The difference between these 2 tests of ToM can be related to different methods of evaluation or different items and contents assessed. A probable interpretation is that girls can understand the social relationships from reading a written text better than interpreting an animated based script.

Based on findings of this study, age did not predict the intentionality ability of the participants. Since the age range of the participants was limited to 9-11 years, it cannot be concluded that age has no effect on the development of understanding intentionality in children. However, in some studies, it has been shown that other components of ToM can be affected by age (21, 30 &^31^). 

The mean score of intentionality among the participants was 29.70+5.88 out of 60. In Abell’s study (2000), the mean mentalizing score in normal children was 1. 73 (SD = 1.03), while the highest probable score was 4. Mohammadzadeh et al (2012) reported the mean intentionality score of 24.98 (SD = 9.82) (min = 2, max = 45) in 7-9-year-old typically developing children. This indicates that the participants responded to 55% of the questions correctly. This percentage reached to 70 in a study on normal adults (Castelli 2000) in which the mean score for intentionality was 15.8 (SD = 1.5) from the total score of 20. With regards to emotional terms, 50 percent of the participants in Mohammadzade et al study used just one term for each animation, while this percent of our participants used 3-6 terms. Our findings showed that IN mean score of 10% of the students in the community was less than 22, indicating that individuals with scores lower than 22 are weak in the detection of IN. In contrast, the students in the upper limit of IN scores had means higher than 37. The participants’ maximum score was 44 and no one reached the maximum score of 60. This test has not been designed for a specific age range; therefore, older children could achieve higher scores. We did not find any other results regarding this paradigm in different age groups to compare with our findings.

## Limitation

The advantages of this study included the recruitment of a substantial number of children studying in the mainstream schools and using all types of the animations from the moving shapes paradigm, while other studies used some of them. However, several limitations should be considered. The intellectual ability of the students was not evaluated. Therefore, we could not correlate the ToM ability to global cognitive function. However, this was not an objective for the study. Second, the participants’ age range was limited to 9-11 years. Therefore, the findings of this study may not be generalizable to other age groups but is generalizable to this age group (9-11 years). Third, due to the cross sectional nature of the study, no causal association could be concluded.

## Conclusion

The moving shapes paradigm was used in a community population of 9-11 year-old children to assess their ability to understand the intentionality of other people’s behaviors and acts. The study showed that this paradigm can be used as a reliable tool in evaluating ToM ability in school-aged children.
